# Identification of two microRNA signatures in whole blood as novel biomarkers for diagnosis of nasopharyngeal carcinoma

**DOI:** 10.1186/s12967-019-1923-2

**Published:** 2019-06-03

**Authors:** Wen Wen, Shi-Juan Mai, Huan-Xin Lin, Mei-Yin Zhang, Jia-Ling Huang, Xin Hua, Chao Lin, Zhi-Qing Long, Zi-Jian Lu, Xiao-Qing Sun, Sai-Lan Liu, Qi Yang, Qian Zhu, Hui-Yun Wang, Ling Guo

**Affiliations:** 1 State Key Laboratory of Oncology in South China, Sun Yat-Sen University Cancer Center, Guangzhou, 510060 People’s Republic of China; 2 Department of Radiotherapy, Sun Yat-Sen University Cancer Center, Guangzhou, 510060 People’s Republic of China; 3 Research Unit of Molecular Epidemiology, Helmholtz Zentrum München, Neuherberg, Germany; 4 Department of Nasopharyngeal Carcinoma, Sun Yat-Sen University Cancer Center, Guangzhou, 510060 People’s Republic of China; 5 Department of Medical Oncology, Sun Yat-Sen University Cancer Center, Guangzhou, 510060 People’s Republic of China

**Keywords:** MicroRNA, Nasopharyngeal carcinoma, Expression profile, Diagnostic signature

## Abstract

**Background:**

Early diagnosis is critical to reduce the mortality caused by nasopharyngeal carcinoma (NPC). MicroRNAs (miRNAs) are dysregulated and play important roles in carcinogenesis. Therefore, this study aimed to identify diagnostically relevant circulating miRNA signatures in patients with NPC.

**Methods:**

Total RNA was extracted from whole blood samples obtained from 120 patients with NPC, 30 patients with head-neck tumors (HNT), and 30 healthy subjects (HSs), and examined by using a custom microarray. The expression levels of four miRNAs identified by using the microarray were validated with quantitative real-time reverse transcription polymerase chain reaction. The 120 patients with NPC and 30 HSs were randomly assigned to training group-1 and validation group-1, respectively. By using significance analysis of microarray (SAM), the specific miRNA expression profiles in whole blood from patients with NPC are obtained. By using lasso regression and adaptive boosting, a diagnostic signature was identified in training group-1, and its accuracy was verified in validation group-1. By using the same methods, another signature to distinguish patients with NPC from those with HNT and HSs was identified in training group-2 and confirmed in validation group-2.

**Results:**

There were 117 differentially expressed miRNAs (upregulated and downregulated fold change ≥ 1.5) between the patients with NPC and HSs, among which an 8-miRNA signature was identified with 96.43% sensitivity and 100% specificity [area under the curve (AUC) = 0.995] to diagnose NPC in training group-1 and 86.11% sensitivity and 88.89% specificity (AUC = 0.941) in validation group-1. Compared with traditional Epstein–Barr virus (EBV) seromarkers, this signature was more specific for NPC. Furthermore, a 16-miRNA signature to differentiate NPC from HNT and HS (HNT-HS) was established from 164 differentially expressed miRNAs, which diagnosed NPC and HNT-HS with 100% accuracy (AUC = 1.000) in training group-2 and 87.04% (AUC = 0.924) in validation group-2.

**Conclusions:**

The present study identified two miRNA signatures for the highly accurate diagnosis and differential diagnosis of patients with NPC from HSs and patients with HNT. The identified miRNAs might represent novel serological biomarkers and potential therapeutic targets for NPC.

**Electronic supplementary material:**

The online version of this article (10.1186/s12967-019-1923-2) contains supplementary material, which is available to authorized users.

## Background

According to the global cancer statistics reported by the International Agency for Research on Cancer, over 84,000 new nasopharyngeal carcinoma (NPC) cases are diagnosed per year, of which more than 80% occur in Asia and 5% in Europe [1]. NPC is highly prevalent in southern China and Southeast Asia, with an annual incidence of 15–50 cases per 100,000 population [2]. It is estimated that in 2015, the incidence and mortality of nasopharyngeal cancer in China were 60,600 and 34,100, respectively [3]. The mortality of NPC has decreased in recent decades because of improvements in treatment technology, especially radiotherapy. Recently, Lv et al. analyzed the data of 7713 patients with NPC obtained from the NCI SEER 17 program released in 2015 and revealed that patients had a 5-year overall survival (OS) of 57.4% in 2000–2007 [4], indicating that the 5-year OS is not satisfactory and should be improved. Furthermore, current treatment decisions depend mainly on the tumor-node-metastasis (TNM) staging system. The common consensus is that stage I NPC is treated by radiotherapy alone, stage II disease by radiotherapy with or without concurrent chemotherapy, and stage III to IV(B) by radiochemotherapy [5, 6]. If patients are diagnosed at an early stage, such as stage I, they only need to receive simple radiation treatment and have a very good prognosis, with few adverse reactions to the therapy. This clearly indicates that early diagnosis is critical to improve the survival and life quality of patients with NPC. Clinically, however, as NPC is asymptomatic or has non-specific symptoms at the early stage, more than 80% of patients with NPC are first diagnosed at a late stage (III or IV), often with cervical lymph node metastasis [7], which leads to reduced survival. Therefore, early diagnosis of NPC is a significant clinical challenge.

Usually, nasopharyngeal biopsy examination and radiological imaging are the most important tools to diagnose NPC, which provide the most definitive evidence to determine the clinical stage, prognosis, and treatment. However, these methods are invasive and painful diagnostic procedures, and are not suitable for early diagnosis or screening NPC in an endemic area. Numerous studies have indicated that the occurrence of NPC is very closely related to Epstein–Barr virus (EBV) infection [8]; therefore, detecting serum anti-EBV antibodies has been used as a non-invasive test to diagnose NPC for more than 40 years [9, 10]. Recently, serum EBV DNA also has been used as a biomarker for NPC diagnosis [11]. However, the etiology and mechanism of how EBV causes this disease are not fully understood [12, 13]. More importantly, the sensitivity and specificity of EBV antibodies are not satisfactory to diagnose NPC. Therefore, other circulating biomarkers have been explored for NPC diagnosis, such as DNA methylation markers [14], fragments of cytokeratin 18 [15], cathepsin B (CTSB) and D (CTSD) [16], insulin like growth factor 1 (IGF-1) [17], arginase 2 (ARG2) [18], autoantibodies against peroxiredoxin 2 (PRDX2) and PRDX3 [19], and long noncoding RNAs [20]. However, these biomarkers require further confirmation in large prospective studies, and currently, none of them have been used in clinical practice. Therefore, new serum biomarkers for the early diagnosis of NPC are urgently required.

MicroRNAs (miRNAs) are a class of small non-coding RNAs of approximately 21–24 nucleotides in length, which lead to the degradation of target mRNAs and/or repression of their translation by complementary binding to their 3′ untranslated region (UTR) [21, 22]. MiRNAs are implicated in various cancers and function as both oncogenes and tumor suppressors in tumor initiation, progression, and response to treatment [23, 24]. Therefore, there are many reports of the use of miRNAs as biomarkers for diagnosis, prognosis, and treatment of cancers. Furthermore, miRNAs can be released from various tumor tissues into circulation during cancer development and progression, indicating the potential of circulating miRNAs as novel noninvasive diagnostic biomarkers for a variety of cancers [25–30]. miRNAs are also aberrantly expressed in NPC tissues [31–33], and serum or plasma miRNAs could be used as biomarkers to diagnose NPC [34, 35].

Recently, miRNAs extracted from whole blood, including plasma and blood cells, have been reported as biomarkers for the early detection of pancreatic cancer [36–38], ovarian cancer [39], lung cancer [40, 41], and gallbladder cancer [42]. miRNAs from whole blood, including white blood cells (neutrophils, eosinophils, basophils, monocytes, and lymphocytes), can be used as diagnostic biomarkers, based on the theory that these circulating white blood cells, as part of the immune system, monitor the patients’ physiological and pathological state and can respond to them by altering their transcriptome [43]. The advantages of whole blood miRNA samples are as follows: (1) A high miRNA yield [44]; (2) it is less error-prone than serum or plasma samples, and (3) the detected miRNAs may originate from plasma, blood cells, circulating tumor cells, tumor tissues, or various organs. Thus, miRNAs from whole blood yield more comprehensive information than those from serum, plasma, or white blood cells [38, 39]. Furthermore, miRNAs in whole blood can originate from distant inflammatory foci. Thus, they are more sensitive in inflammation-related cancers, such as chronic pancreatitis related pancreatic cancer and hepatitis B virus (HBV)-related hepatocellular carcinoma (HCC) [38]. NPC is also considered an inflammation-related cancer [45]. Therefore, whole blood miRNAs should be a better biomarker than serum or plasma miRNAs to diagnose NPC. To the best of our knowledge, there have been no reports on the diagnostic value of whole blood miRNAs in patients with NPC to date.

Early diagnosis of NPC remains the major issue in clinical practice. In this study, a custom miRNA microarray was employed to profile miRNA levels in whole blood from healthy subjects (HSs), patients with NPC, and those with head-neck tumors (HNT). The study aimed to identify non-invasive miRNA signatures to diagnose NPC and to differentially diagnose NPC and HNT with higher accuracy.

## Materials and methods

### Patients and blood samples

Whole blood samples were collected from healthy volunteers termed healthy subjects (HS, n = 30), patients with NPC (n = 120), and those with head-neck tumors (HNT) (n = 30) at the Sun Yat-Sen University Cancer Center during May 2015 to March 2017. Healthy subjects were recruited from those participating in annual physical examination for cancer at the cancer center. The patients were pathologically and clinically diagnosed as NPC or HNT, and staged according to the TNM staging system of International Union against Cancer. The characteristics of all subjects are listed in Table 1. The inclusion criteria and exclusion criteria for patients with NPC are shown in Table 2. None of the patients with HNT were subjected to any treatment, such as surgery, radiotherapy, or chemotherapy, before blood sampling. The Ethical Committees of Sun Yat-Sen University Cancer Center approved this study. Written informed consent was obtained from all participants in the study.

**Table 1:** The clinical characteristics of patients with NPC and controls

Clinical characteristics	NPC (N = 120)	HNT (N = 30)	HS (N = 30)
Age (years)	45.2	55.9	27.1
Sex ratio			
Male	91	24	15
Female	29	6	15
T stage			
1	7	13	
2	19	6	
3	67	8	
4	27	3	
N stage			
0	6	20	
1	26	5	
2	66	5	
3	22	0	
M stage			
0	116	30	
1	4	0	
Clinical stage			
I	0	12	
II	8	5	
III	65	9	
IV	47	4

**Table 2:** The inclusion criteria and exclusion criteria for the patients with NPC

Inclusion criteria	Exclusion criteria
Biopsy-proven, WHO II or III NPC	WHO I NPC or other pathological types
Patients first diagnosed in Sun Yat-Sen University Cancer Center	Patients first diagnosed in another hospital
Patients without any treatment, including radiotherapy, chemotherapy, and surgery	Patients treated by radiotherapy, chemotherapy, and surgery
No second primary tumors	Patients with other tumours

### RNA extraction

Approximately 2–3 ml of whole blood from each donor was drawn into a Blood RNA Preservative Tube and RNA extraction was performed by using an RNA Purification Kit (Norgen Biotek, Thorold, Ontario, Canada) following the manufacturer’s protocol. Briefly, 1.5 ml of RNA Extraction Buffer A and 1.5 ml of RNA Extraction Buffer B were added to the 3 ml of whole blood and mixed well and incubated at − 20 °C for 10 min. Thereafter, the mixture was centrifuged at 4°C at 4000 rpm for 30 min, After discarding the supernatant, 570 μl of Resuspension Solution B and 330 μl of 100% Ethanol were added into the remaining liquid and mixed well. The mixture was then added into a MiniSpin column and the column was centrifuged at 3500 rpm at 4 °C for 1 min. Subsequently, the column was washed three times with 100 μl of Wash Solution A. Finally, 100 μl of Elution Solution A was added into the column and centrifuged at 4 °C at 1000 rpm for 2 min. The RNA concentration was measured by using an ND-1000 spectrophotometer (NanoDrop Technologies).

### Microarray detection

The miRNA microarray was designed and fabricated as described previously [46, 47]. Briefly, the microarray, which was fabricated in our laboratory, contains 1849 probes for miRNA species sourced from the miRBase database (Release 18.0). The RNA (1.0–1.5 μg) samples were labeled with 100 nmol/l of pCp-Cy5 (Jena Bioscience, Germany) and 15 units of T4 RNA ligase (USB) in a total 20 μl of reaction volume at 16 °C overnight. Then, equal volumes of labeled RNA sample and 2× hybridization solution were mixed well and hybridized onto the microarray for 12–18 h at 45 °C. After hybridization, the microarrays were washed in 1× SSC/1% SDS for 10 min at 45 °C, followed by sequential washing in 0.5 × SSC/0.1% SDS solution twice, 0.2 × SSC twice and purified water once for 1 min each time at room temperature. Finally, the microarray was dried in a special small centrifuge and scanned using a LuxScan-10K instrument (CapitalBio, China).

### Gene expression data extraction

The microarray scanning images were digitized by using the GenPix Pro 6.0 program, and the original signal data were extracted. After subtracting the background, the averaged signals of replicated probes were normalized using Quantile normalization. The normalized data were then converted into their log2 values. The microarray data have been deposited in the Gene Expression Omnibus of the National Center for Biotechnology Information (GSE118613).

### Quantitative real-time reverse transcription polymerase chain reaction (qRT-PCR)

For qRT-PCR, total RNA (10 ng) was reverse transcribed into cDNA by using an All-in-One™ miRNA qRT-PCR Reagent Kit (GeneCopoeia) containing Poly A polymerase, a unique Oligo-dT Adaptor primer, and M-MLV Reverse Transcriptase. The quantitative PCR reactions were then carried out with an All-in-One™ qPCR Mix containing SYBR^®^ Green, first-strand cDNA, specific miRNA primers, and the Universal Adaptor PCR Primer (GeneCopoeia) on a PRISM 7900HT system (Applied Biosystems). Every sample was analyzed in triplicate wells, and reactions excluding cDNA were used as negative controls. The thermal cycling conditions were: 95 °C for 10 min for a hot start, and then 40 cycles at 95 °C for 15 s, 60 °C for 20 s, and 72 °C for 10 s. U48 RNA was employed as the endogenous control. The qRT-PCR data were firstly normalized by U48 expression and then by a mean expression value of a given miRNA in the HSs. Therefore, the relative quantification of miRNA expression was presented as 2^−ΔΔCt^.

### Statistical analysis

Student’s t-test and significance analysis of microarray (SAM) were employed to identify the differentially expressed miRNAs (upregulated and downregulated fold changes ≥ 1.5, P < 0.001 and false discovery rate (FDR)-q < 0.05) between the NPC subjects and control subjects (HS and HNT). Lasso regression was then used to screen for the significant miRNAs, which were used to build the diagnostic model for NPC by using Adaptive Boosting. Receiver operating characteristic (ROC) analysis was performed to show the diagnostic efficiency of the 8-miRNA signature and the 16-miRNA signature in the R3.4.0 software.

## Results

### The miRNA expression profiles of whole blood in patients with NPC and those with HNT

**Fig. 1:**
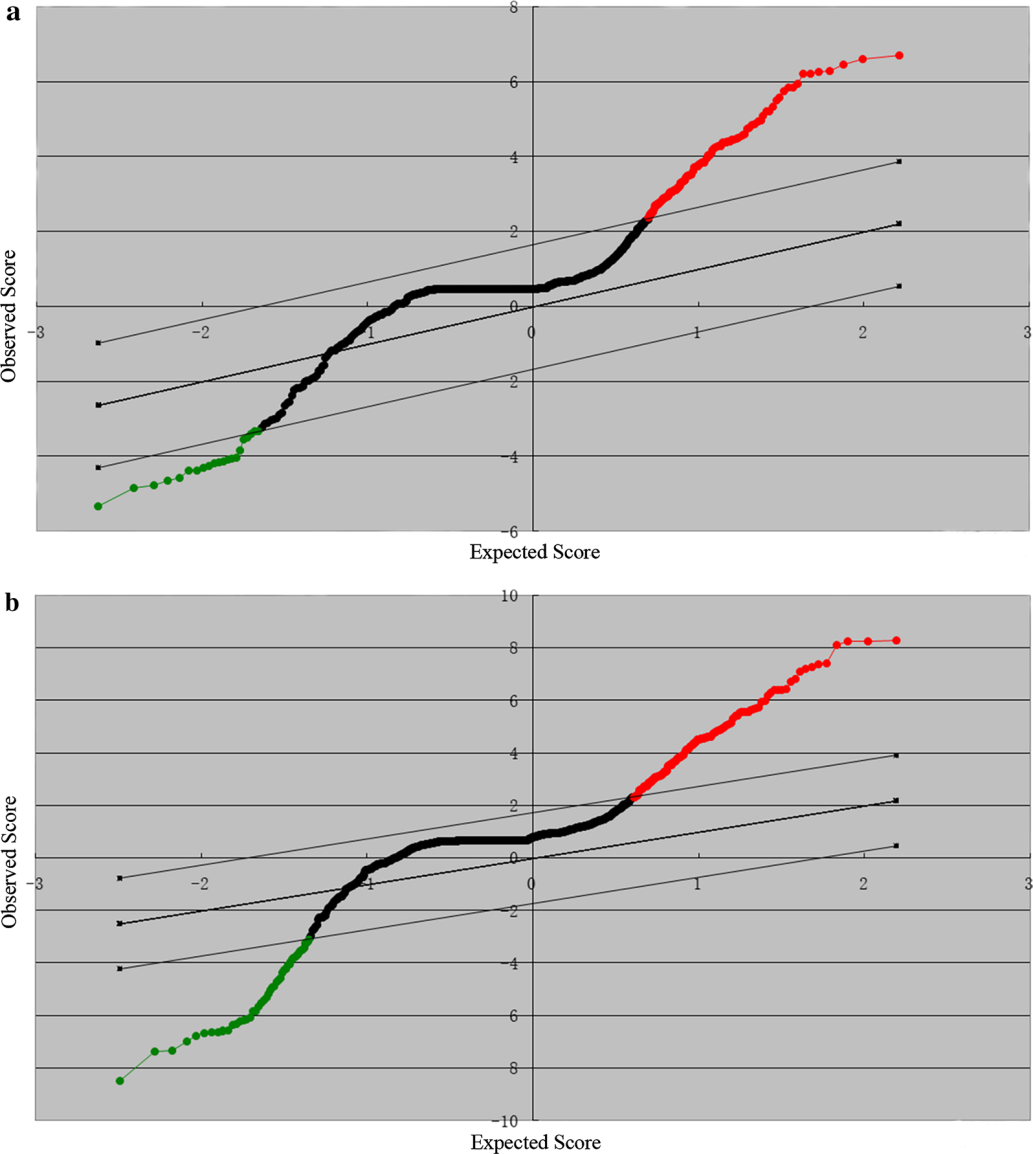
Scatter plot analysis for the microarray expression data of 1849 miRNAs in SAM. Red and green dots represented upregulated miRNA and downregulated miRNA respectively. **a** Scatter plot analysis of miRNA expression between patients with NPC and healthy subjects. **b** Scatter plot analysis of miRNA expression between patients with NPC and the combination of HNTs and HSs

As far as we know, there have been no reports on miRNA expression profiles of whole blood in patients with NPC. In the present study, the miRNA profiles of the whole blood in patients with NPC were first investigated. A total of 150 whole blood samples were collected from 120 patients with NPC and 30 HSs, and total RNA was extracted from these samples. miRNA expression was detected with the custom miRNA microarray. By using SAM software and Student’s t-test, 117 differentially expressed miRNAs (upregulated and downregulated fold change ≥ 1.5) were identified between the patients with NPC and HSs with the FDR set to zero. Among these differential miRNAs, 99 were upregulated and 18 were downregulated in the NPC samples. The SAM plotsheet of miRNAs was presented in Fig. 1a.

In clinical practice, NPC should be discriminated from other HNT patients. In this case, the miRNA profiles between patients with NPC and those with HNT plus the HSs (HNT-HS) were explored. Therefore, whole blood samples from 30 patients with HNT were collected to investigate the miRNA expression by using the microarray. By using the same method as described above, 164 miRNAs that were significantly differentially altered between the NPC group and the HNT-HS group were identified, of which 128 were upregulated and 36 were downregulated in the NPC group. The SAM plotsheet of miRNAs was showed in Fig. 1b. Altogether, two miRNA profiles were identified between the NPC group and HS or HNT-HS group, which would provide the clues to develop diagnostic or differential diagnostic markers, prognostic markers, and therapeutic targets in NPC.

**Fig. 2:**
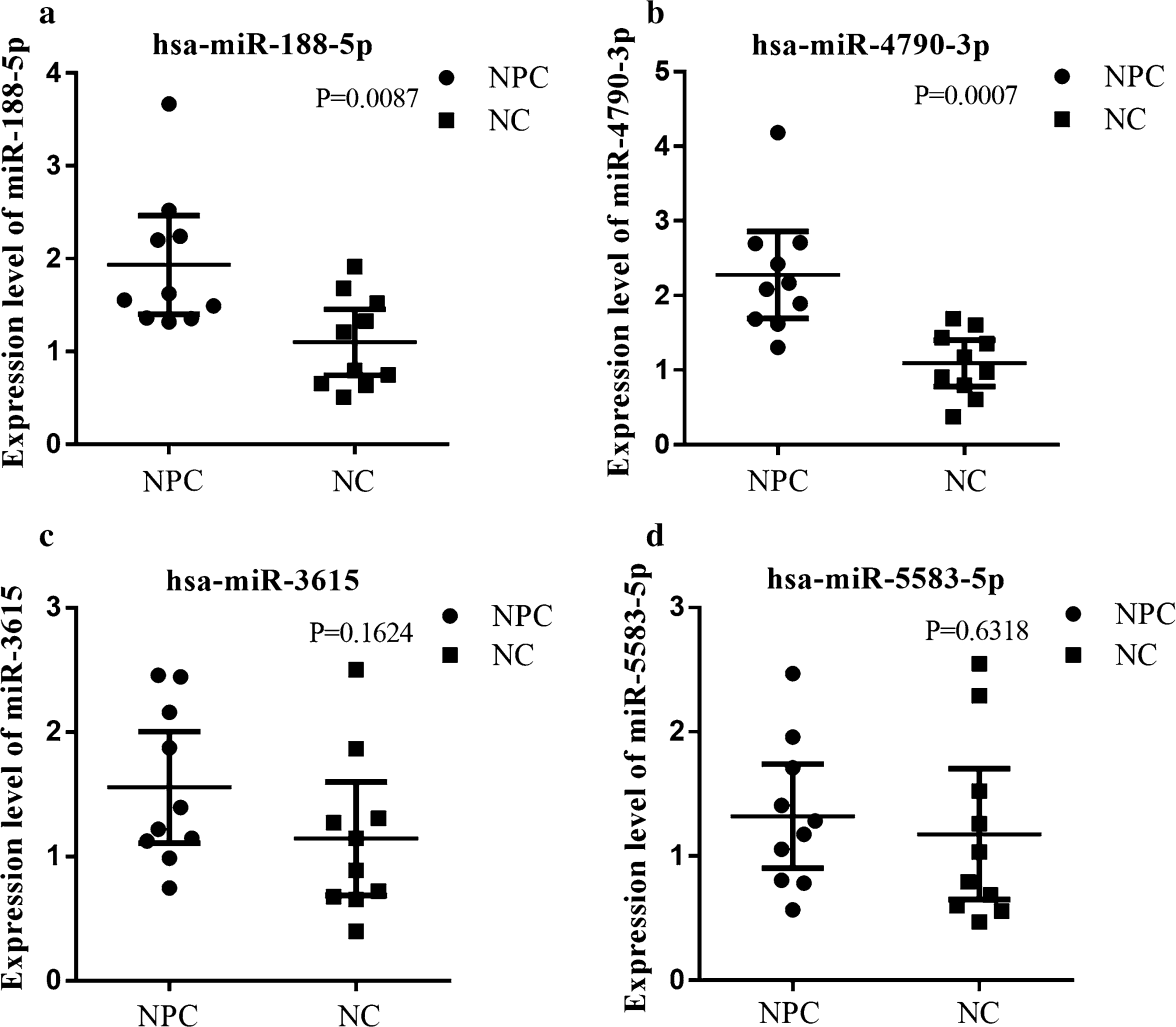
The whole blood expression levels of miRNAs examined by microarray were validated with qRT-PCR in NPC and HS. The expression levels (2^−ΔΔCt^ values) of miR-188-5p (**a**), miR-4790-3p (**b**), miR-3615 (**c**), and miR-5583-5p (**d**) were verified by using quantitative real-time reverse transcription polymerase chain reaction (qRT-PCR) in 20 whole blood samples including 10 HSs and 10 NPC subjects. The whole-blood expression levels of miR-188-5p (**a**), miR-4790-3p (**b**) in the NPC group were significantly higher than those in the HS group (all P < 0.05), and miR-3615 (**c**) and miR-5583-5p (**d**) expression levels were higher in the NPC group than in the HS group but the difference was not significant, which were similar to the results obtained using the microarray in the NPC and HS groups. The qRT-PCR reaction of each sample was performed in triplicate and the mean values were calculated. The expression levels are presented as 2^−ΔΔCt^ value for10 samples in each group

**Fig. 3:**
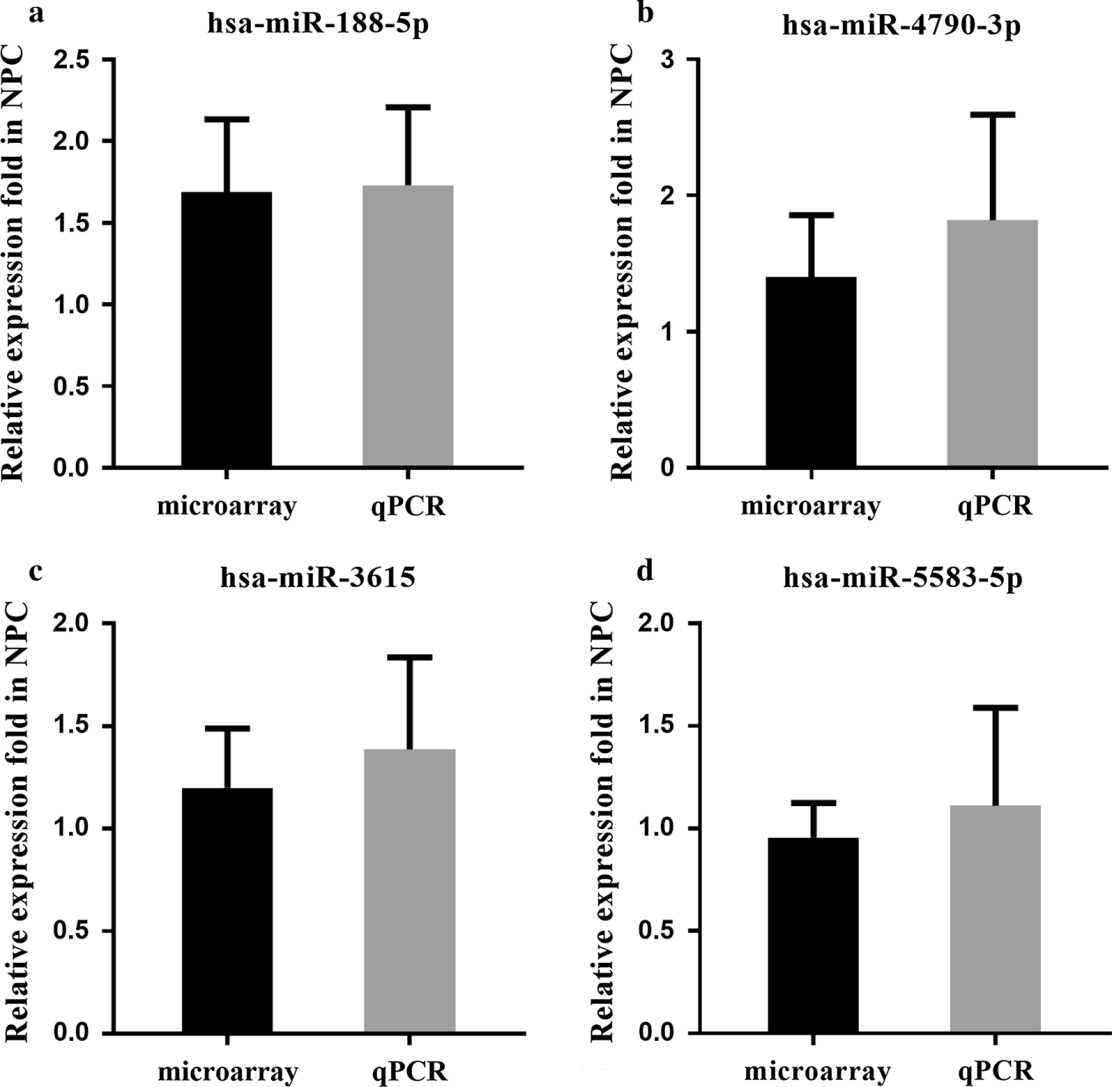
The whole blood relative expression levels of four miRNAs examined by microarray and qRT-PCR are similar in patients with NPC. By taking the mean value of the miRNA expression levels examined by using the microarray (30 samples) and quantitative real-time reverse transcription polymerase chain reaction (qRT-PCR) (10 samples) (2^−ΔΔCt^ values) in HS samples as reference value respectively, the relative fold expression of miR-188-5p (**a**), miR-4790-3p (**b**), miR-3615 (**c**), and miR-5583-5p (**d**) examined by microarray and qRT-PCR in NPC samples were calculated based on the respective reference values and showed no significant differences (all P > 0.05), which indicated that the data obtained by the microarray is reliable

To confirm the reliability of the results from the miRNA microarray, the levels of four randomly selected miRNAs (miR-4790-3p, miR-188-5p, miR-5583-5p, and miR-3615) were measured by using qRT-PCR in randomly chosen blood samples of 10 NPCs and 10 HSs. The qRT-PCR results showed that the levels of miR-4790-3p and miR-188-5p were significantly upregulated in the NPC samples compared with that in the HS samples, and the levels of hsa-miR-5583-5p and hsa-miR-3615 were also higher in NPC samples than in the HS samples but the difference was not significant (Fig. 2). These results were consistent with the results obtained by using the microarray analysis (Fig. 3). This result demonstrated that the data from the miRNA microarray analysis were reliable and could be used for further analysis.

### Identification of an 8-miRNA signature to diagnose NPC in the training group-1 and its verification in the validation group-1

This study’s main aim was to identify a miRNA signature to diagnose NPC. To this end, a total of 150 whole blood samples (120 from patients with NPC and 30 from HSs) were randomly divided into a training group-1, which included 84 NPC samples and 21 HS ones, and a validation group-1 containing 36 NPC samples and 9 HS ones. To build a diagnostic model for NPC, Lasso regression screened out eight significant candidate miRNAs in the training group-1 (Table 3). Then, the microarray expression values of the eight miRNAs in the training group-1 were used to build a diagnostic model to calculate the diagnostic scores by using the AdaBoost machine-learning algorithm in the R 3.4.0 program. A linear combination of M base regressors was constructed and the final boosted model was obtained (the detailed process is described in Additional file 1: Method S1):

$${\text{f(x)}} =\sum\limits^{\text{M}}_{\text{i}=1} \upalpha_{{\text{m}}}\, {\text{G}}_{\text{m}}({\text{x}})$$

**Table 3:** Eight miRNAs identified by Lasso regression in Training Group-1

Variables	Lasso coefficient
(Intercept)	− 8.6460
hsa-miR-188-5p	0.0349
hsa-miR-1908	0.1915
hsa-miR-3196	0.0530
hsa-miR-3935	0.1212
hsa-miR-4284	− 0.2967
hsa-miR-4433-5p	0.1526
hsa-miR-4665-3p	0.1657
hsa-miR-513b	0.4145

In this model, M is 1000. The diagnostic score could be obtained by using this boosted model in the R 3.4.0 program.

**Fig. 4:**
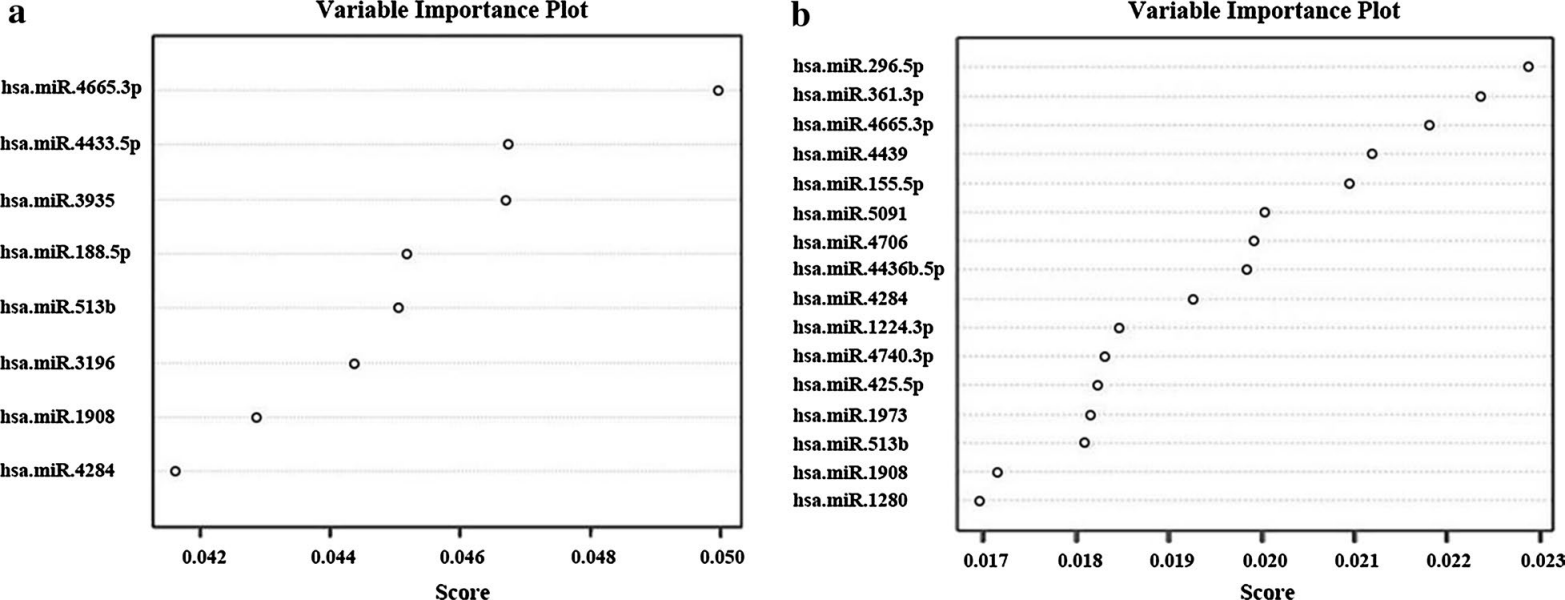
The importance ranking of every miRNA in the two diagnostic signatures. **a** The importance ranking of every miRNA in the 8-miRNA signature in the training group-1. **b** The importance ranking of every miRNA in the 16-miRNA signature in the training group-2. The higher the miRNA score, the greater significance of this miRNA in the auxiliary diagnosis of nasopharyngeal carcinoma

**Fig. 5:**
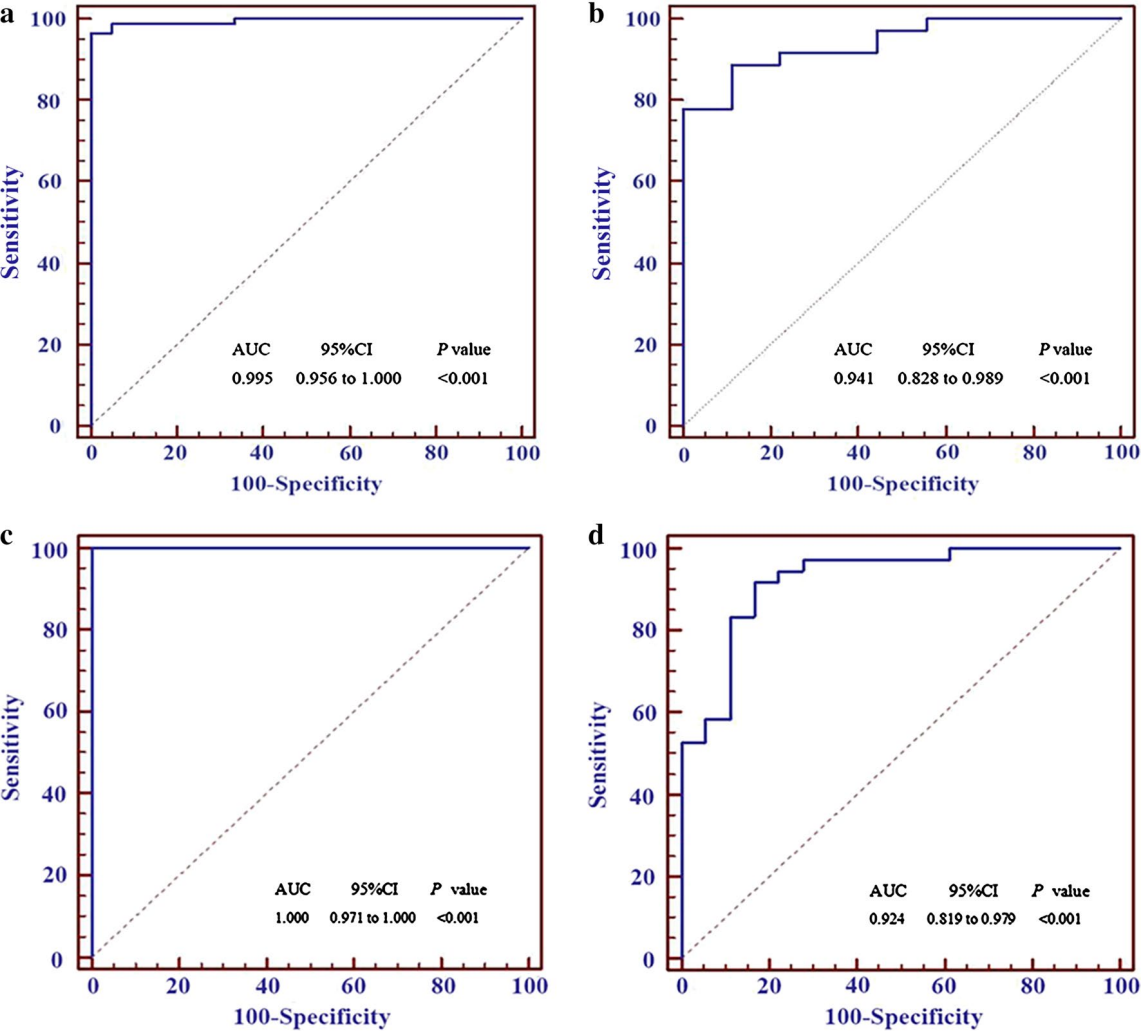
The ROC curves of the 8-miRNA and 16-miRNA signatures. **a** The ROC curve (blue) of the 8-miRNA signature in the training group-1. **b** The ROC curve (blue) of the 8-miRNA signature in the validation group-1. **c** The ROC curve (blue) of the 16-miRNA signature in the training group-2. **d** The ROC curve (blue) of theb16-miRNA signature in the validation group-2

As shown in Fig. 4a, the eight miRNAs were ranked by their roles in the diagnostic model. If the score was greater than or equal to 0.708, the subject was diagnosed as a patient with NPC; otherwise, the subject is diagnosed as a HS. In this way, the 8-miRNA signature diagnosed NPC with 97.14% accuracy (sensitivity: 96.43%, specificity: 100%) in the training group-1 (Table 4 and Additional file 1: Table S1). The ROC analysis showed that the area under the curve (AUC) of the 8-miRNA signature was 0.995, with a P value less than 0.001 in the training group-1 (Fig. 5a).

**Table 4:** The diagnostic results of the 8-miRNA signature in the Training Group-1

Actual diagnosis	The diagnostic results of 8-miRNAs		Total
	NPC N (%)	HS N (%)	
NPC	81 (96.4)	3 (3.6)	84
HS	0 (0.0)	21 (100.0)	21
Total	81	24	105

To verify the diagnostic value of the 8-miRNA signature identified in the training group-1, the reproducibility of the diagnostic signature was further verified in the validation group-1. The result in the validation group-1 indicated an accuracy of 86.67% (sensitivity: 86.11%, specificity: 88.89%) (Table 5 and Additional file 1: Table S2). The ROC analysis revealed that the AUC of this signature is 0.941 in the validation group-1 (P < 0.01) (Fig. 5b). The results indicate this 8-miRNA signature is a powerful and reproducible biomarker to diagnose NPC.

**Table 5:** The diagnostic results of the 8-miRNA signature in the Validation Group-1

Actual diagnosis	The diagnostic results of 8-miRNAs		Total
	NPC N (%)	HS N (%)	
NPC	31 (86.1)	5 (13.9)	36
HS	1 (11.1)	8 (89.9)	9
Total	32	13	45

### Identification and Verification of the 16-miRNA signature to differentiate NPC from other head-neck tumors and healthy subjects in the second training and validation groups

The differential diagnosis of NPC and other head-neck tumors is difficult in some cases; therefore, an miRNA signature was sought that could differentiate NPC from HNT. Whole blood samples from 30 patients with HNT were collected in the same cancer center. In the clinic, physicians simply need to determine if the patient has NPC or not. Therefore, the 30 patients with HNT were combined with the 30 HSs mentioned above as a control group (the HNT-HS group). The 180 samples (120 NPCs, 30 HNTs, and 30 HSs) were also randomly divided into a training group-2 (including 84 NPCs, 20 HNTs and 22 HSs) and a validation group-2 (containing 36 NPCs, 10 HNTs, and 8 HSs). Lasso regression identified 16 miRNAs from 164 differentially expressed miRNAs for the diagnosis of patients with NPC in comparison with the HNC-HS group (Table 6). Then, the expression values of the 16 miRNAs were used to construct another diagnostic model to compute the diagnostic scores for all subjets by using AdaBoost machine-learning algorithm in the R 3.4.0 software. The rankings of the roles of the 16 miRNAs in this 16-miRNA diagnostic signature are shown in Fig. 4b. With 0.5 as the cutoff value, if the diagnostic score of a subject was larger or equal to 0.5, the subject would be diagnosed as having NPC; otherwise the subject would belong to the HNT-HS group. In this way, this diagnostic model yielded a 100% accuracy rate (100% sensitivity and 100% specificity) in the training group-2 (Table 7 and Additional file 1: Table S3). Similarly, the ROC curve analysis showed that the AUC of the 16-miRNA signature was 1.000 (P < 0.01) (Fig. 5c).

**Table 6:** Sixteen miRNAs identified by Lasso regression in the Training Group-2

Variables	Lasso coefficient
(Intercept)	− 5.5820
hsa-miR-1224-3p	0.2305
hsa-miR-1280	− 0.0086
hsa-miR-155-5p	− 0.2466
hsa-miR-1908	0.0121
hsa-miR-1973	− 0.0849
hsa-miR-296-5p	0.1406
hsa-miR-361-3p	0.0403
hsa-miR-425-5p	0.0401
hsa-miR-4284	− 0.3668
hsa-miR-4436b-5p	− 0.2057
hsa-miR-4439	0.0300
hsa-miR-4665-3p	0.2091
hsa-miR-4706	0.1847
hsa-miR-4740-3p	0.1901
hsa-miR-5091	− 0.1342
hsa-miR-513b	0.5499

**Table 7:** The diagnostic results of the 16-miRNA signature in the Training Group-2

Actual diagnosis	The diagnostic results of 16-miRNAs		Total
	NPC N (%)	HS + HNT N (%)	
NPC	84 (100.0)	0 (0.0)	84
HS + HNT	0 (0.0)	42 (100.0)	42
Total	84	42	126

To further determine the diagnostic capacity of this signature, we calculated the diagnostic score for each subject using the expression value of 16 miRNAs in validation group-2 with the 16-miRNA diagnostic signature model established in training group-2. The result showed that the 16-miRNA diagnostic signature model led to 94.44% sensitivity and 72.22% specificity for NPC diagnosis in validation group-2 (Table 8 and Additional file 1: Table S4). At the same time, the ROC analysis showed that the AUC of the 16-miRNA signature was 0.924 (P < 0.01) (Fig. 5d). The results showed that the 16-miRNAs could correctly distinguish the patients with NPC from the HSs and HNT patients.

**Table 8:** The diagnostic results of the 16-miRNA signature in the Validation Group-2

Actual diagnosis	The diagnostic results of 16-miRNAs		Total
	NPC N (%)	HS + HNT N (%)	
NPC	34 (94.4)	2 (5.6)	36
HS + HNT	5 (27.8)	13 (72.2)	18
Total	39	15	54

## Discussion

With progress in high-throughput examination techniques for miRNAs, an increasing number of circulating miRNAs have been observed to be correlated with tumor diagnosis, progression, prognosis, and treatment response, demonstrating that these miRNAs have a large potential for diagnosis, prognosis, and treatment in patients with tumors [33, 48]. The circulating miRNAs with altered level have been proposed as promising biomarkers with several advantages, including stability in body fluid samples, non-invasive sampling, and easy and rapid manipulation. However, published data about the peripheral blood miRNA profiles in patients with NPC are limited.

Zeng et al. [35] reported that four miRNAs (miR-17, miR-20a, miR-29c, and miR-223) were expressed differentially in the serum of 20 patients with NPC compared with that in 20 non-cancerous controls. Prediction of NPC by using the 4-miRNA-based diagnostic equation with a new Ct difference method gave 97.3% sensitivity and 96.5% specificity. In the study of Zheng et al. [49], miR-548q and miR-483-5p were identified as potential biomarkers of NPC by comparing the plasma miRNA profiles of 31 patients with NPC and 19 controls. The combination of these two miRNAs obtained 67.1% sensitivity and 68.0% specificity for NPC diagnosis. Liu et al. [50] identified that miR-22, miR-572, miR-638, and miR-1234 were differentially altered in the serum of patients with NPC, and the 4-miRNA signature may add prognostic value to the TNM staging system. Wang et al. [51] used next-generation sequencing to verify that the plasma level of miR-483-5p, miR-103, and nmiR-29a could be helpful to predict survival in patients with NPC.

Previously, we identified a 5-miRNA signature including miR-93, miR-142-3p, miR-29c, miR-26a, and miR-30e as an independent prognostic factor of NPC from 312 paraffin-embedded NPC specimens by using a custom microarray containing 873 miRNA probes in 2012 [32]. The total number of miRNAs has increased substantially in recent years; therefore, 1849 probes for miRNAs were designed according to miRBase database (Release 18.0) and applied in the present study. In this study, 117 differentially expressed miRNAs were found between patients with NPC and HSs, 99 of which were upregulated and 18 were downregulated in patients with NPC in the training group-1. The finding that miRNAs upregulated in whole blood outnumbered those that were downregulated in patients with NPC was consistent with a previous report that miRNA activity is globally increased in cancers [52].

In recent decades, many reports have shown that the development and progression of NPC is a strongly associated with the human herpesvirus, Epstein–Barr virus (EBV). Studies have shown that patients with NPC have high levels of a broad spectrum of EBV antibodies [53–55]. Detection of a combination of IgA antibodies against EBV capsid antigen (VCA/IgA) and early antigen (EA/IgA) by using an immunofluorescence assay has been established as a standard tool for NPC screening [56]. However, the sensitivity and specificity of the combination of two IgA antibodies were 50.9% and 95.2%, respectively, indicating lower sensitivity. This might be reflect the fact that EBV is a member of the herpesvirus family and causes persistent infection in more than 90% of adults [57, 58]. Moreover, EBV infection is involved in other hematological and epithelial malignancies, such as Hodgkin’s lymphoma, Burkitt’s lymphoma, and gastric carcinoma [8, 53, 59, 60].

In the present study, there was a considerable difference in ages between the patients with cancer and the HSs. Although it may have some impact on miRNA expression levels, we believe that the influence is small and not significant. For example, in the study of Muñoz-Culla et al. in 2017, blood leucocytes of 38 healthy subjects aged from 24 to 79 years old were detected with 847 miRNA probes, and 35 miRNAs showed ≥ 1.5-fold change [61]. However, only has-miR-1280 in the 16-miRNA signature is one of these 35 miRNAs, indicating that the miRNA level changes related to ages would have a very limited impact on miRNA expression profiling in patients with NPC.

The 8-miRNA signature was identified with a high diagnostic value for distinguishing NPC from HSs (96.43% sensitivity and 100% specificity in the first training group and 86.11% sensitivity and 88.89% specificity in the first validation group). Compared with traditional seromarkers, this 8-miRNA signature has a much higher sensitivity for NPC. During 2006 and 2010, only 4.5% (120/2671) of patients with NPC in the Sun Yat-Sen University Cancer Center had stage I–II disease, suggesting that early diagnosis is a major challenge. In the present study, the proportion of early stage patients (including stage I and II) was 6.7% (8/120), which was similar to the 4.5% detected in 2006 to 2010. All eight patients with early stage disease were diagnosed correctly by using the 8-miRNA signature. Therefore, the identified 8-miRNA signature will be of significant clinical utility to improve the early diagnosis of NPC and improve patient survival.

HNTs are common carcinomas worldwide and have been linked with EBV. Reports show that EBV seromarkers and products can be detected in tumors originating from the tonsil, parotid gland, nasal fossa, soft palate, oropharynx, supraglottic larynx, and the base of the tongue [62, 63]. Therefore, EBV seromarkers are not specific for differentiating NPC from other EBV-related head-neck tumors. To better differentially diagnose NPC and HNT, we identified a 16-miRNA diagnostic signature that could distinguish NPC from HNT and HSs. The 16-miRNA signature reached 100% accuracy in the training group-2 and 87.04% in the validation group-2. To the best of our knowledge, this is the first report of whole-blood miRNA signatures for the diagnosis and differential diagnosis of NPC and HNT, which have potential clinical applications in NPC diagnosis.

The present study has some restrictions and limitations. First, the samples only come from a single medical center; second, the sample number is not large enough for extensive statistical analysis; third, geographically, all of the patients live in South China where NPC is pervasive. Therefore, before the two miRNA signatures could be used in clinical practice, the analysis of further independent samples from different regions is required to verify the findings. In addition, the function and mechanism of these miRNAs in the carcinogenesis of NPC are not clear. For example, miR-1908, which is upregulated in the whole blood of patients with NPC and is a component of the two signatures, has been reported to repress the PTEN tumor suppressor pathway and functions as an oncogene in glioblastoma [64], which suggests that this miRNA may also play an important role in NPC development and progression. Consequently, it will be important to explore the levels of the proteins targeted by the miRNAs in the both signatures and their possible roles in NPC carcinogenesis. Furthermore, monitoring the dynamic changes in whole-blood miRNA levels might be beneficial for the early diagnosis of NPC in high-risk populations, and for the treatment, recurrence, and metastasis in patients with NPC.

## Conclusion

In the present study, we profiled miRNA expression levels in the whole blood of patients with NPC and identified two miRNA signatures (8-miRNA and 16-miRNA signatures) with high diagnostic accuracy for NPC, HS, and HNT-HS subjects. The 8-miRNA and 16-miRNA signatures are promising and potentially powerful biomarkers for the diagnosis and differential diagnosis of NPC, and are the first reported diagnostic signatures for NPC identified from whole blood samples. In addition, to the best of our knowledge, the 16-miRNA signature is the first meaningful diagnostic signature to differentiate NPC from head-neck tumors and healthy subjects. Moreover, the role and mechanism of the miRNAs in the two signatures in the development, progression, and immunoreactivity of NPC should be explored in a future study. Further multicenter prospective studies are warranted to validate these diagnostic signatures for NPC.

## Additional file

**Additional file 1.** Additional Methods and Tables.

## Data Availability

The datasets supporting the conclusions of this article are included within the article (and its additional files)

## Abbreviations

NPC: nasopharyngeal carcinoma
miRNAs: microRNAs
HNT: head-neck tumor
OS: overall survival
TNM: tumor-node-metastasis
EBV: Epstein–Barr virus
SAM: significance analysis of microarray
qRT-PCR: quantitative real-time reverse transcription polymerase chain reaction
HBV: hepatitis B virus
HCC: hepatocellular carcinoma
HS: healthy subject
RPM: revolutions per minute
ROC: receiver operating characteristic
AUC: area under the curve
FDR: false discovery rate
