# Supplementary material for: Identification of two microRNA signatures in whole blood as novel biomarkers for diagnosis of nasopharyngeal carcinoma
Source: J Transl Med. 2019 Jun 3;17:186. doi: 10.1186/s12967-019-1923-2 (PMC6547589; doi:10.1186/s12967-019-1923-2)
Supplement: Supplementary file 1 — Additional file 1. Additional Methods and Tables. [file 12967_2019_1923_MOESM1_ESM.docx]

**Additional Materials**

**Identification of two microRNA signatures in whole blood as novel biomarkers for diagnosis of nasopharyngeal carcinoma**

Wen Wen, et al.

**1. Additional methods**

**2. Additional Tables**

**1. Additional methods**

The workflow of the Adaboost algorithm can be depicted as follows:

Step 1 Uniformly initialize the weight distribution of the training data.

Step 2 Train m-th base regressor Gm on a dataset with a weight distribution of Dm.

Step 3 Calculate the mean squared prediction error of Gm denoted by em.

Step 4 Calculate the coefficient of Gm, which is a measure of confidence in the predictor.

$\alpha_{m}=\frac{1}{2}\mathrm{In}\frac{E^{2}-e_{m}}{e_{m}} , E=sup\left\{ |y_{i}-\hat{y_{i}}| \right\} ,i=1,2,\ldots，N$

Step 5 Update the weight distribution of training dataset Dm+1, making it a new distribution.

$$D_{m+1}=(W_{m+1,1},\ldots,W_{m+1,i},\ldots,W_{m+1,N})$$

$$W_{m+1,i}=\frac{W_{\mathrm{mi}}}{Z_{m}}\exp\left\{ -\alpha_{m}y_{i}G_{m}(x_{i}) \right\},i=1,2,\ldots,N$$

$$Z_{m}=\sum_{i=1}^{N} W_{\mathrm{mi}}\exp\left\{ -\alpha_{m}y_{i}G_{m}(x_{i}) \right\}$$

Step 6 Form=1, 2,…, M, repeat Steps 2 to 5 until M base regressors are trained.

Step 7 Construct a linear combination of M base regressors and obtain the final boosted model.

$$f\left( x \right)=\sum_{i=1}^{M} \alpha_{m}G_{m}(x)$$

In our model, M is 1000. The diagnostic score can be obtained by using this boosted model in the R 3.4.0 program .

**2. Additional Tables**

**Table S1. The diagnostic efficacy of the 8-miRNA signature in Training Group-1**

| Diagnostic efficiency | 8-miRNA signature |
| --- | --- |
| Sensitivity (%) | 96.43 |
| Specificity (%) | 100.0 |
| Accuracy (%) | 97.14 |
| Positive predictive value (%) | 100.0 |
| Negative predictive value (%) | 87.5 |

**Table S2. The diagnostic efficacy of the 8-miRNA signature in Validation Group-1**

| Diagnostic efficiency | 8-miRNA signature |
| --- | --- |
| Sensitivity (%) | 86.1 |
| Specificity (%) | 88.9 |
| Accuracy (%) | 86.7 |
| Positive predictive value (%) | 96.9 |
| Negative predictive value (%) | 61.5 |

**Table S3. The diagnostic efficacy of the 16-miRNA signature in Training Group-2**

| Diagnostic efficiency | 16-miRNA signature |
| --- | --- |
| Sensitivity (%) | 100.0 |
| Specificity (%) | 100.0 |
| Accuracy (%) | 100.0 |
| Positive predictive value (%) | 100.0 |
| Negative predictive value (%) | 100.0 |

**Table S4. The diagnostic efficacy of the 16-miRNA signature in Validation Group-2**

| Diagnostic efficiency | 16-miRNA signature |
| --- | --- |
| Sensitivity (%) | 94.4 |
| Specificity (%) | 72.2 |
| Accuracy (%) | 87.0 |
| Positive predictive value (%) | 87.2 |
| Negative predictive value (%) | 86.7 |
